# Investigating the Role of Perceived Information Overload on COVID-19 Fear: A Moderation Role of Fake News Related to COVID-19

**DOI:** 10.3389/fpsyg.2022.930088

**Published:** 2022-06-17

**Authors:** Chong Zhang, Tong Cao, Asad Ali

**Affiliations:** ^1^School of Public Security Management, People’s Public Security University of China, Beijing, China; ^2^School of Communication, Hankou University, Wuhan, China; ^3^Department of Electrical Engineering, Foundation University, Islamabad, Pakistan

**Keywords:** COVID-19, information overload, fake news, social media, COVID-19 fear

## Abstract

During crises and uncertain situations such as the coronavirus disease 2019 (COVID-19) pandemic, social media plays a key function because it allows people to seek and share news, as well as personal views and ideas with each other in real time globally. Past research has highlighted the implications of social media during disease outbreaks; nevertheless, this study refers to the possible negative effects of social media usage by individuals in the developing country during the COVID-19 epidemic lockdown. Specifically, this study investigates the COVID-19 fear using the survey data collected from a developing country. In total, 880 entries were used to analyze the COVID-19 fear using the AMOS software. Findings indicated that information-seeking and sharing behavior of individuals on social media has a significant impact on perceived COVID-19 information overload. Perceived COVID-19 information overload has a positive impact on COVID-19 fear. In addition, fake news related to COVID-19 strengthens the relationship between perceived COVID-19 information overload and COVID-19 fear. The implication and limitations of the study are also discussed in the final section of the study.

## Introduction

The coronavirus disease 2019 (COVID-19) outbreak, which began in China in December 2019 and spread across the globe (Farooq et al., 2020), has been identified as a significant epidemic issue. The number of infected people and deaths caused by COVID-19 continues to dominate media attention on multiple platforms, including news, radio, print, and social media, with the lead headlines typically focusing on the number of infected people and deaths (Farooq et al., 2020; Liu et al., 2021). These incidents tend to have psychological consequences on individuals all over the globe. Specifically, the use of social media became the primary source of information regarding the COVID-19 outbreak (Farooq et al., 2020; Apuke and Omar, 2021). Scholars reported that the use of social media during the epidemic lockdown has affected the wellbeing of individuals (Apuke and Omar, 2021) but has considerable benefits for participants (Khan et al., 2019a; Pitafi et al., 2020c; Cao et al., 2021). In addition, studies indicated that social media is widely used to exchange risk-related information including flooding, terrorism, the COVID-19 epidemic, and earthquake (van Dijl et al., 2019). Previous studies indicated that people use various social media tools to spread and seek information related to crises (West, 2015; Pitafi et al., 2018b; Han and Ciravegna, 2019; Islam et al., 2020), but some are true and others are fake. Social media is a public platform that provides an opportunity for an individual to seek, share, and post COVID-19-related information (Shah et al., 2019). Indeed, social media has been used extensively to collect health details since the COVID-19 epidemic (Nabity-Grover et al., 2020). An enormous quantity of health information presents challenges for individuals to identify, compile, and properly handle valuable information. This wide-ranging use of social media contributes to information overload, implying that users of social media are exposed to information but lack the capability to absorb it (Chen and Wei, 2019). Previous studies have found that over-explosion of infection-related details may escalate people’s fear of that infection (Farooq et al., 2020; Gever et al., 2021). Specifically, extraordinary scenarios like infection epidemics posts on social media may cause millions of individuals to panic. Consistent with past studies, this study investigates the information-seeking and sharing behavior of individuals on social media in COVID-19 fear through perceived COVID-19 information overload.

As the COVID-19 pandemic began spreading across the world, various media including social and print media reported its symptoms and effects (Hopp et al., 2020). Google search patterns showed a sharp rise in COVID-19 requests, peaking in February 2020. The rapid spread of information and rumors has resulted in an explosion of COVID-19-related disinformation, prompting the WHO to develop a news tracker to identify and reject repeatedly circulated false news (Farooq et al., 2020). Although false news is not new, it has become more worrying as a result of the prevalence of social media, which allows further interaction and the advent of novel ideas (Khan et al., 2019b). As a result, people on social media may advance concepts or distribute news by posts (Pitafi et al., 2020a; Cao et al., 2021), comments, or re-tweets; therefore, they are inevitably influential and exposed to uncontrollable content, particularly news written by independent authors. According to studies, posting content on social media has become quick and easy; individuals use this forum to keep relatives, friends, and others up to date on important topics that may concern their lives (Nabity-Grover et al., 2020; Apuke and Omar, 2021). The more people who post news, the more possible it is that they would share false news if they are not careful about the material. Spreading such disinformation not only causes fear, unpleasant feelings, information overload, or even online illegal acts that are detrimental to people (Allcott and Gentzkow, 2017; Di Domenico et al., 2021) but also makes it difficult to utilize relevant informative material on social media effectively. In this vein, fake news related to COVID-19 on social media may strengthen the relationship between perceived COVID-19 information overload and COVID-19 fear.

**FIGURE 1:**
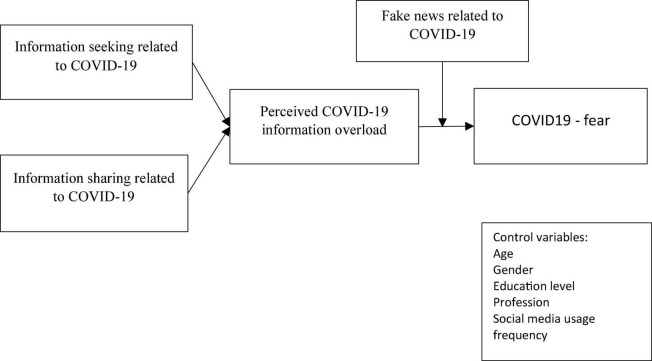
Proposed model.

The objective of this study is to investigate the fear of the COVID-19 outbreak using data collected from the developing region community. Based on communication visibility theory, the conceptual model of this study explores the usage of social media by the developing region community during COVID-19 (refer to Figure 1 for conceptual model). Communication visibility theory suggested that posts and articles on social media platforms are easily viewed and exchanged by individuals (Leonardi, 2014; Wu et al., 2020). This study has several theoretical and practical implications. First, according to the author’s literature review, this study is the first study conducted in a developing region to investigate the role of social media during the COVID-19. Second, this study also highlighted the role of social media in the context of communication visibility theory. Previous scholars have examined the workplace use of social media by applying communication visibility theory (Chen and Wei, 2019; Pitafi and Ren, 2021). Third, this study contributes to the negative impact of social media literature by investigating the COVID-19 fear and moderating the role of fake news related to COVID-19. Fourth, in developing regions, COVID-19 outbreak is at its peak, and individuals may also be benefited from this study.

## Theoretical Background and Hypothesis Development

### Theory of Community Visibility

Social media is an interactive public platform that enables people to share, connect, and collaborate without regard for time or space (Leonardi, 2014; Wei et al., 2020). On social media, communication has no longer remained private, and all communications are visible to all the parties (Leonardi, 2014; Chen and Wei, 2019). Social media is a digital technology making people’s behaviors, decisions, and preferences more visible (Ali et al., 2019a). It reduces the amount of effort, and individuals can view and share information easily, thus making information more visible. In the context of social media, scholars have determined communication visibility as the extent to which other user communications and posts are easily available on social media (Treem et al., 2020). Visibility referred to how individuals’ presence, activity, and communication were made noticeable to other users (Leonardi, 2014). Recent literature indicates that visibility is a distinctive characteristic of social media, differentiating it from other communication and collaboration technologies (Treem and Leonardi, 2013; Chen and Wei, 2019). Prior research studies on visibility have yielded significant results and greatly advanced our understanding (Leonardi, 2014; Chen and Wei, 2019; Sun et al., 2020; Pitafi and Ren, 2021), but are limited to workplace communication. This limitation inhibits researchers from reaching a greater understanding of how visibility shapes users’ processes and interactions; as such, there is a compelling need to further explore the meaning of visibility. Social media facilitates users’ ability to visualize the posts, comments, and responses on other users’ posts with whom they have never communicated. Compared with traditional news media, fake or inaccurate information about COVID-19 is circulated more on social media platforms including Twitter and Facebook.

In the context of this study, communication visibility of social media suggested how people use social media technology to share and seek COVID-19-related information. If an individual does not know what information exists on social media, they will not spend time attempting to find it. To contribute to communication visibility theory, this study explores how people reciprocate perceived COVID-19 content posted by others within their social network with specific social media communicative features. In the context of the COVID-19 outbreak, we contended the nature of visibility provided by social media, through which people have multiple options for presenting or accessing posts, and blogs, related to COVID-19 and making them accessible to other users.

### Use of Social Media for Crisis

Social media has become ubiquitous and is often used for crisis management to disseminate information. It is a web-based platform where individuals emote and react instantly (Houston et al., 2015; Nadeem et al., 2021). Scholars reported that several posts exist on social media platforms whenever any major event occurs (Evers et al., 2020). Social media allows both specialists and the general population to easily disseminate information to a massive number of people. However, the studies related to social media and crisis management are mixed (Zubiaga et al., 2016; Han and Ciravegna, 2019). For example, Han and Ciravegna (2019) argued that social media posts are useful in emergency services and can remotely identify areas affected by crises. Social media users post reports on what they are seeing and hearing. Saroj and Pal (2020) also investigated the relationship between natural disasters and social media usage and found that posts on social media may help the individual or government authorities to minimize the damages. Further, scholars argued that social media contains information that could enhance the situational awareness of the local public during the crisis (Hiltz et al., 2011; Zubiaga et al., 2016). On the contrary, some studies also reported that most individuals are often reluctant to use social media, as the information on social media is unverified (Farooq et al., 2020; Latif et al., 2020). Allcott and Gentzkow (2017) also reported that social media usage plays a critical role in the spread of fake news. Han and Ciravegna (2019) also discussed the rumor related to crisis management on social media. Social media tools like Facebook offer several ways including posting news, sharing videos and articles, and updating the status in which an individual can interact (Kanwal et al., 2019; Pitafi et al., 2020a). In addition, social media amplifies the spread of news, and people read news through links shared on social media.

Scholars of information systems also warned about the negative impact of social media use and enquire more research (Cao and Yu, 2019; Chen and Wei, 2019; Anser et al., 2020; Raza et al., 2020), especially social media’s impact on fake news. For example, social media is the main source of fake news, and fake news rapidly spread among large population through social media. Fake news can exist anywhere and be mediated through the same channels as any other news, and social media, in particular, has been found to accelerate the spread of fake news. For example, fake news circulated on social media that toilet roll paper will be out of stock due to factory closures in China (Cato et al., 2021). As a result, toilet roll paper sales enhance 134.4% in February and were out of stock in some countries by the end of March. Similarly, another study conducted by Shin et al. (2018) explores the political rumors being circulated through Twitter after the 2012 United States referendum, which were compiled from January 2012 to January 2013. According to the report, rumor has the potential to cause ripples and reappear several times after its original release, whereas accurate information rarely appears again (Cao et al., 2021; Liu et al., 2021). The abundance of unclear, ambiguous, and inaccurate information during COVID-19 led to information overload and accelerated COVID-19 fear. In this setting, perceived COVID-19 information overload and its connection to COVID-19 fear may reveal further insights into human behavior on social media. Specifically, the impact of increased social media use and growing pandemic information on social media during the pandemic lockdown has been subject to limited investigation. Against this specific backdrop, this study investigates the impact of COVID-19-related information seeking and sharing behavior of individuals on COVID-19 fear through perceived COVID-19 information overload.

### COVID-19

The novel coronavirus infection (COVID-19) was first discovered in Wuhan, Hubei Province of China (Zhong et al., 2020; Iqbal and Younas, 2021). The COVID-19 virus spread quickly across the world, causing social anxiety and worries in several areas (Özmen et al., 2021). At the time of writing, the COVID-19 epidemic had affected over a million people worldwide. In response to the rapid and massive amount of cases both inside and outside China, the World Health Organization (WHO) announced public health emergency on January 30, 2020 (Iqbal and Younas, 2021). The WHO named the virus a pandemic on March 11, 2020, and it was confirmed that the virus had expanded to 203 countries by March 30, 2020. The most specific indications of the illness include headache, weakness, sore throat, muscle discomfort, and breathlessness (Pakpour and Griffiths, 2020). According to statistics, the COVID-19 epidemic circulated rapidly across the world. It has now infected 218 countries and territories, with a record of more than 59.2 million reported incidents, and has resulted in more than 1.4 million recorded deaths worldwide.

The existence of COVID-19 in the developing region was reported on February 27, 2020, by government authorities, and the root of the infection was traced back to Iran (Iqbal and Younas, 2021). On the same day, another case was reported by the federal government of the developing region. After 15 days, the overall number of reported cases (COVID-19 positive) crossed 20 out of 471 suspected cases, with the Sindh Province having the largest number, followed by Gilgit Baltistan (Iqbal and Younas, 2021). All reported cases had recently traveled to Iran, Syria, or London. In response to such outbreaks, every country has implemented various control mechanisms, for example, overall lockdown and partial lockdown based on an assessment of strict control on their citizens’ movement. The developing region government has taken various successful steps to monitor the transmission of COVID-19. These actions entail the initial closure of public parks, educational schools, industry, and tourism. The present government of the developing region has implemented a specific policy initiative known as smart lockdown, which has been appreciated by WHO representatives (Chandir et al., 2020). In addition, according to the Director of WHO, COVID-19 was not only a worldwide pandemic but also an “infodemic,” exposing serious problems caused by a flood of disinformation and fake news regarding COVID-19 on media, especially on social media, which intensified COVID-19 anxiety (Iqbal and Younas, 2021). Therefore, the aim of this study was to explore the COVID-19 fear-related factors including individual use of social media and fake news.

### Information Seeking and Perceived COVID-19 Information Overload

According to the literature, information seeking is described as the extent to which information is exchanged in social media technologies that can provide individuals with necessary and up-to-date information (Tsai and Kang, 2019; Apuke and Omar, 2021). Social media is the primary mode of communication for individuals seeking and sharing information for professional or personal reasons (Graffigna et al., 2017; Lai et al., 2021). Specifically, during a disaster, people tend to use social media to seek and share information since users can quickly access social media from everywhere. Scholars indicated that several telecommunications networks are unable to cope with a rapid spike of calls, thousands of individuals attempting to call at the same time or text messages directly after a disaster event (Han and Ciravegna, 2019; Saroj and Pal, 2020); however, social media platforms such as Facebook and Twitter will serve and update the public. Social media is widely used to exchange risk-related information such as flooding, terrorism, and earthquake (Ryan, 2013; Nand et al., 2019; van Dijl et al., 2019). For example, Ryan (2013) discovered that the primary reason people used social media during a flood in Australia was to seek and share information about their families and friends, as well as the actual state of the flood and its potential consequences. In fact, in the case of a natural disaster, where power is shut off or disconnected, communication networks (radio/television) may often be interrupted. Even so, people can access social media through mobile phones, tablets, and other devices for real-time information sharing and seeking. In the context of this study, information overload is an outcome of social media use during the COVID-19.

During COVID-19, the usage of social media grew dramatically. Individuals continue to connect with relatives, colleagues, and employers in the COVID-19 region and enquire about their protection and health. Information seeking and sharing have been associated with the use of social media for COVID-19 news dissemination. One potential explanation for this sharing and seeking news on social media is that individuals can easily participate in the creation and dissemination of information. Furthermore, social media was often used to collect health information during the outbreak of COVID-19, either supplementing or eliminating traditional sources. Such a large amount of health information induces information fatigue, which makes it impossible for individuals to efficiently process and manage the necessary useful information (Cao et al., 2019; Cao et al., 2021). This extensive use of social media results in information overload, which means that users are exposed to information that exceeds their capacity to consume it (Pitafi et al., 2018a; Chen and Wei, 2019). When people fail to absorb and manage the amount of information provided on social media, the problem of information overload occurs (Pitafi and Ren, 2021). According to recent research, information overload is exacerbated by the individual’s communications, content dissemination, messages, likes, updates, shares, and posts or re-tweets on social media (Molinillo et al., 2019). Furthermore, factors such as social interaction, entertainment, and seeking social status may also motivate the individual to seek and share information. As a consequence, we believe that as people’s movement is limited due to the COVID-19 epidemic, and as cases of the epidemics rise, individuals will seek and exchange information on social media without too much consideration. They may also exchange such information across their networks, potentially increasing information overload. Furthermore, governments have imposed bans on human movements and travel, numerous workplaces have closed, and most citizens are at home (Farooq et al., 2020; Bilal et al., 2022); they have more time to seek and share information related to COVID-19. Drawing from this point of view, we contended that, owing to the already millions of posts on COVID-19 on social media, seeking and sharing information is resulting in COVID-19 information overload. Based on the above evidence, this study proposes the following hypotheses:

H1a: On social media platforms, information seeking related to COVID-19 has a positive effect on perceived COVID-19 information overload.

H1b: On social media platforms, information sharing related to COVID-19 has a positive effect on perceived COVID-19 information overload.

### Perceived COVID-19 Information Overload and Fear of COVID-19

Fear is an adaptive reaction to the existence of danger that mobilizes resources to cope with a possible threat. The COVID-19 epidemic is still unknown and underway, and people have expressed fear about coronavirus’ potential danger. According to Asmundson and Taylor (2020), the COVID-19 outbreak has triggered widespread uncertainty and has caused global instability. COVID-19 was identified at the end of 2019 in Wuhan, China, during this phase, and the individual’s daily life routine changed; one major change brought by COVID-19 was COVID-19 fear. Specifically, COVID-19 has been linked to high-speed infection and a high death rate, and several people have shown fear of the virus. Previous research has shown that overexposure to infection-related information may increase people’s fear of that infection (Mertens et al., 2018). Given the unknown future of COVID-19, social media applications are widely used by individuals to share and update information related to COVID-19. For example, App Annie reports indicate that the total time per day consumed on social media has increased by 20% since the start of 2020. In addition, on March 24, 2020, Facebook revealed a 50% increase in messages during the COVID-19 pandemic. The indiscriminate use of social media may increase the perception of information overload.

Social media users are exposed to a large quantity of information, which can cause information overload (Chen and Wei, 2019; Pitafi and Ren, 2021). Specifically, during crisis events, social media is the primary source of information. According to recent studies, the massive amount of COVID-19 information generated on social media has exhausted users and had a significant effect on their psychological wellbeing (Islam et al., 2021; Liu et al., 2021; Li et al., 2022). The social media platform contains several articles and posts about the COVID-19 epidemic, including frightening figures, helpful suggestions, and gallows humor about the coronavirus (Pitafi et al., 2020a; Liu et al., 2021). The regular updates on social media about COVID-19, such as affected cases, death ratios, and preventive measures, have heightened people’s fear of the COVID-19. Furthermore, details on social media about social threats including the economic crisis, resource shortages, educational loss, and the health of family members caused by COVID-19 create more fear (Liu et al., 2021). As the level of misinformation regarding COVID-19 on social media enhanced, individuals were likely to become overloaded with pandemic details, which in turn will increase COVID-19 fever among individuals. The enormous amounts of complex information related to COVID-19 overwhelmed the individuals’ information-processing capability, inhibited their ability to establish an unbiased analysis of COVID-19, and strengthened the higher level of fear related to the coronavirus epidemic. As a result of the discussion, this research proposes the following hypothesis:

H2: On social media platforms, perceived COVID-19 information overload has a positive effect on COVID-19 fear.

### Moderating Role of Fake News Related to COVID-19

Fake news is a systemic crisis of humans that causes stress, fear, and confusion in human civilization. It is not new on the Internet; but, due to social media, the dissemination has reached new and unparalleled heights. Although user-generated posts and well-developed sharing features of social media have enhanced connectivity and information exchange (Pitafi et al., 2019), it has also increased fake news. Instead of delivering relevant and accurate information, the dissemination of fake news on social media could quickly enter a wide scale (Rashid et al., 2020), causing unnecessary misunderstanding, information overload, and fear among individuals. For example, in the 2013 Boston Marathon bombing, false news circulated on Twitter; although explanations became accessible within hours, one of the rumors circulated from 40 tweets to over 4,000 in 10 min. Duffy et al. (2020) described fake news as any content that resembles a real news story but contains inaccurate and misleading information. Fake news poses a serious threat to public health during pandemics such as the COVID-19. The rapid spreading of such fake news largely occurs through social media, which could increase the fear of COVID-19. In this study, fake news is described as inaccurate content such as misconceptions, misinformation, fake stories, falsehoods, and misleading or incorrect content about the COVID-19 epidemic that is deliberately or accidentally disseminated on social media platforms. Nevertheless, there is a difference between the creation and dissemination of fake posts on social media platforms (Egelhofer and Lecheler, 2019). Fake news dissemination might be accidental, but its formation may be strongly intentional. This viewpoint is connected to the COVID-19 problem, where people can re-share inaccurate content to assist others. Therefore, this research investigates the moderating impact of fake news related to COVID-19 in the relationship between perceived information overload and COVID-19 fear.

The COVID-19 epidemic is experiencing another related crisis caused by fake news on the social media platform, which mainly disrupts individual health communication and reinforces widespread fear. According to recent studies that in recent months, most of the fake news shared on social media platforms was related to COVID-19 (Pennycook et al., 2020). For example, Rodríguez et al. (2020) found that people on social media post and spread false news rather than evidence-based news regarding COVID-19. In addition, details of fake news on social media including the economic crisis, death ratio, resource shortages, employment loss, and the health of family members caused by COVID-19 create more fear (Rashid et al., 2022). Several reasons may lead to increased information overload in the situation of the COVID-19 epidemic. First, the scenario of COVID-19 was novel, which forces individuals to obtain and share new information on a social media platform. Second, the problem of COVID-19 began suddenly and presented a serious health danger, prompting individuals to adapt and exchange new information rapidly. Third, people all over the world posted their views related to COVID-19 on social media, and there was a constant stream of news, some true and some false. The amount of information made it more complicated to interpret the true condition of the COVID-19. The resultant uncertainty led to increased information overload for social media users. In a novel situation like COVID-19, fake news is being presented at high volumes, and the human cognitive capacity gets overloaded. Therefore, on the basis of the above literature support, this study proposes the following hypothesis:

H3: Fake news related to COVID-19 on social media platforms moderates the relationship between perceived information overload and COVID-19 fear. Such that the higher the fake news-related to COVID-19 on the social media platform, the stronger the relationship between perceived COVID-19 information overload and COVID-19 fear.

## Research Methodology

### Data Collection Procedures

Our target population was social media users, who use Facebook and WhatsApp for communication, interaction, information seeking, and sharing with their friends, family, and colleagues. We used the snowball sampling methodology in an online questionnaire survey to collect data from social media users in a developing region. This survey was conducted in a developing region due to the increasing popularity of social media. Additionally, the objective of our study is to find implications of social media for knowledge seeking and sharing about COVID-19, and how such information is related to motivating social media users to engage in social distancing as a protective measure against virus spread. Thus, the target population must have experience in using Facebook and WhatsApp and must be living in a society affected by COVID-19. Thus, conducting a study in a developing region is expected to yield interesting findings, extending the studies, which are mostly conducted in other western countries and China. We searched university groups and invited local university students to their involvement in increasing data collection by sharing an online survey. These students were offered a small amount of money in return for their sharing of an online questionnaire with their social media contacts on Facebook and WhatsApp. In 15 days of deployment, we received 339 valid responses, after discarding incomplete responses, invalid responses (same answers to all questions), and people who do not have experience of using two social media platforms. Table 1 reveals the demographic statistics of the sample respondents.

**TABLE 1:** Sample demographics.

		Frequency	Percentage
Gender	Male	179	52.8
	Female	160	47.2
Age range	Less than 20 years	15	4.4
	21–30 years	194	57.2
	31–40 years	98	28.9
	41 years and above	32	9.4
Education level	High school	15	4.4
	Bachelors degree	118	34.8
	Masters degree	164	48.4
	Doctor degree	42	12.4
Profession	Student	80	23.6
	Government employed	107	31.6
	Private employed	78	23.0
	Self-employed	74	21.8
Social media usage	Hourly	169	49.9
	Once per day	113	33.3
	Several times per day	48	14.2
	Several times per week	9	2.7

### Instruments

Previous research has suggested to develop a questionnaire based on available measures of the constructs to increase the validity of the study (Khan and Ali, 2018; Ali et al., 2019b; Islam et al., 2021; Ma and Wang, 2021). Accordingly, the survey measures were adapted from the existing literature to increase the validity of the results. All items were measured on 7 points Likert scales from strongly disagree (1) to strongly agree (7). We used seven items adopted from Apuke and Omar (2021) to measure intentions to seek COVID-19 information. A sample item of this measure is “I will seek help and suggestions using social media in decision making during COVID-19 situation.” Eight items were used to measure intentions to share COVID-19 information. A sample item of this measure is “I share content related to COVID-19 on social media to disseminate information that might interest or entertain others.” These items were adopted from the previous study (Apuke and Omar, 2021). Perceived COVID-19 information overload was measured by using three items adopted from Liu et al. (2021). A sample item of this measure is “I cannot handle all the COVID-19-related information on social media effectively.” We used three items to measure fear of COVID-19 adopted from Vrinten et al. (2014) and Liu et al. (2021). A sample item of this measure is “I get worried when I find out my friends are having fun without me during lockdown.” Finally, we used five items to measure fake news related to COVID-19. A sample item of this measure is “I share content on social media related to COVID-19 without checking facts through trusted sources.”

Considering previous studies on social media and COVID-19 (Islam et al., 2020; Masood et al., 2021; Ali et al., 2022), we controlled variables including age, gender, education level, profession, and social media usage frequency to generate more robust results.

## Analysis and Results

Following the two-step approach suggested by Anderson and Gerbing (1988). The two-step approach is widely adopted by information systems scholars (Bao et al., 2020; Wang et al., 2022). Following the relevant literature, this study initially examines the measurement model to check the reliability and validity of the data and then tests the structural model to test the significance of the established hypotheses (Bahadur et al., 2019; Masood et al., 2021).

### Measurement Model

The confirmatory factor analysis was conducted to assess the validity of the measures according to previous studies (Ali et al., 2020; Islam et al., 2020; Khan et al., 2020; Ali et al., 2021a,b,c). The CFA results show a satisfactory model fit [χ2 = 512.33, df = 281, goodness of fit index (GFI) = 0.90, comparative fit index (CFI) = 0.97, Tucker–Lewis index (TLI) = 0.96, and root mean square error of approximation (RMSEA) = 0.05]. In addition, factor loadings (ranging between 0.58 and 0.95) were all above the satisfactory range (Urbach and Ahlemann, 2010). Furthermore, convergent validities of the measures are assessed through Cronbach’s alpha, average variance extracted, and composite reliabilities. Table 2 reveals that Cronbach’s alpha values ranging from 0.82 to 0.94 are well above 0.70 threshold. Values of AVE range between 0.58 and 0.80 and are also above the satisfactory value of 0.50. The values of composite reliabilities range between 0.82 and 0.94 and are also reasonable. Together, these values confirm the convergent validity of the measurement model. Following the suggestion of Bagozzi et al. (1991), we assessed the discriminant validities of the measures by calculating the square roots of the AVE, which have to be greater than correlations with other constructs. Table 3 reveals that the square roots of AVE are greater than related correlations, thus indicating a satisfactory discriminant validity of the measures. As Table 4 shows, the items loaded well on their corresponding constructs than their cross-loadings onto other constructs. Thus, these values indicate a good discriminant validity of the measurement model. Taken together, the above analysis identifies that our measure model adequately satisfies the validity and reliability statistics and thus guarantees to test the structural model of the study.

**TABLE 2:** Results of the confirmatory factor analysis.

Construct	Items	Cronbach’s alpha	Composite reliability	Average variance extracted
Intentions to seek Information related to COVID-19	7	0.94	0.94	0.69
Intentions to share Information related to COVID-19	8	0.92	0.92	0.58
Perceived COVID-19 information overload	3	0.82	0.82	0.61
COVID-19 fear	3	0.92	0.92	0.80
Fake news related to COVID-19	5	0.94	0.94	0.75

**TABLE 3:** Descriptive statistics and correlations matrix.

Construct	Mean	Std. deviation	1	2	3	4	5	6	7	8	9	10
1. Gender	0.47	0.50	–									
2. Age	2.43	0.72	−0.06	–								
3. Education level	2.69	0.74	−0.20**	0.16**	–							
4. Profession	2.43	1.08	0.01	−0.04	−0.01	–						
5. Social media usage frequency	1.70	0.81	0.03	−0.10	−0.03	−0.05	–					
6. Intentions to seek Information related to COVID-19	4.61	0.76	0.01	0.06	0.05	−0.01	−0.02	**0.83**				
7. Intentions to share Information related to COVID-19	4.74	0.64	0.08	0.13*	0.04	−0.06	0.03	0.37**	**0.76**			
8. Perceived COVID-19 information overload	5.43	1.15	0.00	0.11*	0.04	−0.08	−0.03	0.22**	0.19**	**0.78**		
9. COVID-19 fear	3.80	1.12	0.11*	0.13*	0.05	−0.10	−0.10	0.17**	0.13*	0.30**	**0.89**	
10. Fake news related to COVID-19	4.07	0.65	0.03	0.07	0.02	−0.10	0.09	0.41**	0.45**	0.13*	0.05	**0.87**

*p = 0.05, **p = 0.01, diagonal cells represent square roots of average variance extracted. Bold diagonal cells show Cronbach’s alpha values.

**TABLE 4:** Item loadings and cross-loadings.

		Factor				
Construct	Items	1	2	3	4	5
1. Intentions to seek Information related to COVID-19	SEE1	**0.68**	0.00	0.13	0.01	−0.03
	SEE2	**0.89**	−0.01	−0.02	−0.01	−0.04
	SEE3	**0.75**	0.08	0.06	0.03	−0.01
	SEE4	**0.84**	0.03	0.00	−0.01	0.07
	SEE5	**0.89**	−0.06	−0.02	−0.03	0.01
	SEE6	**0.87**	0.01	−0.06	0.04	0.01
	SEE7	**0.89**	0.00	−0.05	−0.02	−0.02
2. Intentions to share Information related to COVID-19	SH1	0.08	**0.65**	−0.04	−0.07	0.10
	SH2	−0.03	**0.88**	−0.02	0.01	−0.07
	SH3	−0.11	**0.93**	0.00	−0.02	0.01
	SH4	−0.05	**0.85**	−0.07	0.02	0.01
	SH5	0.01	**0.79**	0.04	0.01	0.05
	SH6	0.06	**0.63**	0.11	−0.03	−0.02
	SH7	0.06	**0.76**	0.05	0.01	−0.07
	SH8	0.07	**0.58**	−0.03	0.05	0.00
3. Perceived COVID-19 information overload	OV1	−0.06	0.03	−0.07	0.05	**0.86**
	OV2	0.01	0.07	0.06	0.03	**0.70**
	OV3	0.05	−0.10	0.05	−0.06	**0.78**
4. COVID-19 fear	CF1	−0.01	−0.01	0.02	**0.92**	−0.04
	CF2	0.07	0.01	−0.01	**0.88**	0.01
	CF3	−0.05	−0.01	0.00	**0.88**	0.05
5. Fake news related to COVID-19	FA1	−0.01	0.01	**0.79**	−0.02	0.07
	FA2	0.03	0.06	**0.84**	−0.02	0.04
	FA3	0.00	−0.02	**0.95**	0.03	−0.05
	FA4	−0.01	0.00	**0.95**	0.02	−0.04
	FA5	−0.02	−0.04	**0.82**	0.00	0.01

*Highlighted values represent loadings of items on their corresponding variables.*

All data in our study were reported by a single respondent at one point in time, which is prone to common method bias (Podsakoff et al., 2003). We employed two methods to assess the potential effects of common method bias. First, Harmen’s single factor test reveals that the first factor did not account for the majority of variance (34.86%). This test reveals that common method bias is not an issue in our data (Bahadur and Ali, 2021). Second, we conducted two CFA tests and compared the fit indices. The results reveal that the hypothesized five-factor model better fits the data (χ2 = 512.33, df = 281, RMSEA = 0.05, GFI = 0.90, CFI = 0.97, and TLI = 0.96) than the alternative single-factor model in which all constructs were combined into a single factor (χ2 = 3612.72, df = 291, RMSEA = 0.18, GFI = 0.47 CFI = 0.52, and TLI = 0.47) (Ali et al., 2022). Moreover, significant moderating effects also mitigate the possibility of common method bias (Siemsen et al., 2010). Thus, together, our two methods and significant moderating effects show that the possibility of common method bias is insignificant in our study.

### Structural Model

#### Hypothesis Testing

The SPSS Amos 24.0.0 software was used to test the direct and mediating hypotheses of this study. Results are presented in Table 5. Specifically, social media platforms used for information seeking related to COVID-19 has a positive effect on perceived COVID-19 information overload (β = 0.26, SE = 0.09, *p* < 0.01), supporting hypothesis H1a. Results also show that social media used for information sharing related to COVID-19 has a positive effect on perceived COVID-19 information overload (β = 0.24, SE = 0.10, *p* < 0.05), and thus, H1b is supported. Furthermore, hypothesis H2 predicted that perceived COVID-19 information overload has a positive effect on COVID-19 fear. Information-seeking behavior during COVID-19 on social media is positively related to social distance, and thus, H2 is supported (β = 0.28, SE = 0.05, *p* < 0.001). These results provide support for direct hypothesized relationships (H1a, H1b, and H2) of this study.

**TABLE 5:** Path analysis results of the structural model.

Hypothesized relationships	β	S.E.	Significance level	Conclusion
H1a: Intentions to seek information related to COVID-19 to perceived COVID-19 information overload	0.26	0.09**	0.01	Supported
H1b: Intentions to share information related to COVID-19 to perceived COVID-19 information overload	0.24	0.10*	0.05	Supported
H2: Perceived COVID-19 information overload to fear of COVID-19	0.28	0.05***	0.00	Supported
**Moderation hypothesis**				
Fake news related to COVID-19	0.03	0.05***	0.00	
H3: Interaction effect	0.10	0.05*	0.05	Supported
**Control variables:**				
Age to fear of COVID-19	0.14	0.08	0.07	
Gender to fear of COVID-19	0.28	0.11**	0.01	
Education level to fear of COVID-19	0.06	0.08	0.42	
Occupation to fear of COVID-19	−0.08	0.05	0.14	
Social media usage frequency to fear of COVID-19	−0.12	0.07	0.09	

*p = 0.05, **p = 0.01, and ***p = 0.001. Interaction effect = Fake news related to COVID-19 × perceived COVID-19 information overload.

### Moderation Analyses

We used Model 1 of PROCESS macro in SPSS to test the moderating effects of fake news related to COVID-19 on social media platforms on the relationship between perceived information overload and COVID-19 fear. In line with the predictions, fake news related to COVID-19 on social media platforms moderates the relationship between perceived information overload and COVID-19 fear (β = 0.10, SE = 0.05, *p* < 0.05).

**FIGURE 2:**
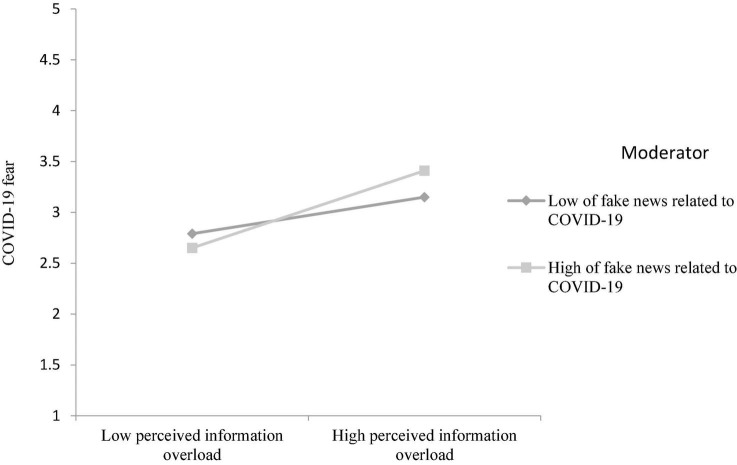
Interaction effect of fake news related to COVID-19 on social media platforms and perceived information overload on COVID-19 fear.

Following prior recommendations (Aiken et al., 1991), we plotted the relationship between perceived information overload and COVID-19 and COVID-19 fear at high (1 SD above mean) and low (1 SD below mean) levels of fake news related to COVID-19 on social media platforms (see Figure 2). As shown in Figure 2, the relationship between perceived information overload and COVID-19 and COVID-19 fear was stronger at high value of fake news related to COVID-19 on social media platforms (β = 0.38, SE = 0.07, *p* < 0.001) than at low value of fake news related to COVID-19 on social media platforms (β = 0.17, SE = 0.07, *p* < 0.05). These results provide further support to our moderating hypotheses H3.

## Discussion, Implications, and Limitations

### Discussion

This study aims to predict COVID-19 fear among social media users in a developing region using primary data. Specifically, this study measured the role of COVID-19-related information seeking and sharing behavior of individuals on COVID-19 fear through perceived CVOID-19 information overload. The outcome indicated that the COVID-19-related information seeking and information sharing behavior of individuals has a significant impact on perceived COVID-19 information overload, supporting H1a and H1b. Recent results also indicated that governments have imposed lockdown, several workplaces have closed, and individuals most of the time are at home (Farooq et al., 2020); they have enough time to seek and share COVID-19 information on social media. In addition, previous studies’ results indicated that most individuals share COVID-19 epidemic posts on social media for enjoyment, socializing, and self-presentation purposes (Nabity-Grover et al., 2020; Apuke and Omar, 2021), which also increases the COVID-19 information overload. The results also indicate that the perceived COVID-19 information overload on social media increased the fear of COVID-19, validating H2. The massive amounts of information related to COVID-19 on social media exceeded the information-processing capacity of the individuals and further limited their ability to understand the COVID-19 scenario, which led to a higher level of COVID-19 epidemic fear. Prior studies also indicated that perceived COVID-19 information overload heightened fear of COVID-19 in the young generation (Liu et al., 2021).

In addition, results also show that fake news related to COVID-19 strengthens the significant relationship between perceived COVID-19 information overload and COVID-19 fear. H3 is also validated by this study and related previous studies (Kim and Dennis, 2019; Pennycook et al., 2020). Specifically, the local government has imposed a lockdown, and individuals are using social media applications most of the time to establish social connections with relatives and friends, posting COVID-19 posts, cures, and preventive steps, which contributes to the dissemination of fake news articles. This amount of fake posts further strengthens the COVID-19 information overload and leads to COVID-19 fear among individuals.

### Theoretical Contribution

Based on the results, this study contributes to existing theoretical literature in several ways. First, this study focused on the COVID-19 epidemic and social media usage. Specifically, this research study focused on the negative side of social media use and contributes to the existing literature on the dark side of social media usage in the context of the COVID-19 epidemic (Cao and Sun, 2018; Pitafi et al., 2020b). Our findings are important and timely which indicates the social media use by individuals in the circumstance of a worldwide epidemic of COVID-19. However, social media became the main information source during the COVID-19 emergency lockdown for some group of individuals, and the effectiveness of social media use as a means of communication during such events remains less evident (Mertens et al., 2020). Second, a previous study has investigated the impact of perceived COVID-19 information overload on COVID-19 fear (Liu et al., 2021), but did not explore the factors of perceived COVID-19 information overload. The findings of this study indicated the significant impact of COVID-19 information seeking and sharing behavior on perceived COVID-19 information load.

Third, based on communication visibility theory, current results indicated that COVID-19 information overload has a significant impact on COVID-19 fear. Previous study results also indicated that individuals seek and share posts on social media platforms without verification for enjoyment and self-promotion (Farooq et al., 2020), which causes COVID-19 fear among the population.

Fourth, results also indicated that COVID-19-related fake news on social media strengthens the relationship between perceived COVID-19 information overload and COVID-19 fear. Previous literature has examined the COVID-19 fake news as an outcome construct (Apuke and Omar, 2021), but did not investigate the impact of COVID-19 fake news on individual psychology. However, the results of this study indicated that fake news related to COVID-19 on social media platforms strengthens the relationship between perceived COVID-19 information overload and COVID-19 fear. Finally, the contribution of this study is that almost all fake news research is largely focused on the Western part of the world. Contrary to this, we used samples from a developing region, which has gained less research attention thus far.

### Practical Contribution

This study sets up some practical implications. First, this study has shown that individual motivations for COVID-19 information sharing and information seeking create perceived COVID-19 information overload. We recommend social media users be cautious when posting content, even though it is done to assist others. In general, our research indicates that the developing region community spreads unverified information about the COVID-19, which can contribute to the dissemination of fake news, creating fear among people and harm to their wellbeing. Second, results also indicated that perceived COVID-19 information overload has a significant impact on COVID-19 fear. The positive impact of COVID-19 information overload on COVID-19 fear can be explained by the fact that information overload does not allow an accurate understanding of the situation at hand.

Third, our study results indicated that fake news related to COVID-19 strengthens the relationship between perceived COVID-19 information overload and COVID-19 fear. Numerous fake news circulated on social media during COVID-19 cause several citizens to take unexpected action, like damaging 5 G cellular network towers (Ahmed et al., 2020). According to Kim and Dennis (2019), one approach to minimize the dissemination of disinformation is to encourage people to pay attention to media sources. Developing region is a developing country with no social media access policy, unlike other countries such as China. As a result, we suggested that the government enact certain social media regulations to address fake news.

Finally, there is an immediate need to properly consider the dissemination of fake news and cope with this negative aspect of social media. Moreover, we discovered that social media is being utilized for information sharing and seeking, which has increased the perceived information overload related to the COVID-19 outbreak. As a practical approach to preventing the dissemination of fake news on social media, we propose to restrict the time spent on social media and access news in detail from trustworthy channels. Social media designers can create certain social media policies for individual users. For example, a social media designer can limit the number of posts posted or submitted by people, as well as the time of each user.

### Limitation

Although this research has several theoretical and practical implications, we also mention some limitations for future research. We conducted this study with a focus on the COVID-19 pandemic and drew our sample from the developing region population. It is possible that our findings may not be generalized to general fake news sharing. Nonetheless, the findings may be generalized to other nations that have a similar culture to developing regions like India, Nepal, and Bangladesh. Second, Facebook is a popular social media application in a developing region (Sharif et al., 2021); future scholars conduct a similar study and only consider Facebook. Future researchers could extend this study to explore another context to validate the outcome of this research.

Third, the limitation of this study is related to the procedure of data collection. We collected cross-sectional responses from developing region social media users during the COVID-19 pandemic. While we ensure the validity and reliability of our data, some geographical, cultural, and contextual specificity may affect the outcome. Another issue related to the sample is that our sample is mixed; however, the young generations are addictive users of social media (Pitafi et al., 2020a). Hence, we recommended that future scholars may focus on the young generation, and this may create a more interesting outcome.

Fourth, our research did not measure whether cultural context, age, employment, experience, or gender could moderate the impact of fear of COVID-19. The use of social media may vary related to age, experience, and education levels of individuals. For example, individuals aged over 55 years and educated tend to choose traditional news sources such as television (Alexander, 2014). Future researchers should include these demographic factors and analyze whether it has some impact on the results of this model. Previous scholars also reported that lack of experience, trust in online information, and laziness in conforming to the information sources promote the individuals in seeking and sharing fake news (Talwar et al., 2019). Furthermore, we recognized that the samples of this study are small in comparison with the total population of the developing region, which could have influenced the predictors of our independent and moderating variables; nevertheless, the sample size is satisfactory and acceptable (Pitafi and Ren, 2021). Future studies may expand the sample size to obtain a more accurate statistical result.

Finally, we did not include the mediating construct in this study and used fake news related to COVID-19 as a moderator. Furthermore, we have only concentrated on the negative effects of social media; nevertheless, throughout COVID-19, individuals may find social media use beneficial. As a consequence, future researchers should examine the positive effect of social network use on the COVID-19 epidemic (Leonardi, 2015). Scholars can use the mediating construct and use another moderator variable to produce an interesting outcome.

## Conclusion

This study aimed to examine the impact of information sharing and seeking behavior of individuals on COVID-19 fear through perceived COVID-19 information overload. In addition, this study also highlighted the moderating role of fake news related to COVID-19, which strengthens the relationship between perceived COVID-19 information overload and COVID-19 fear. As social media is an open and public platform, all the individuals share and exchange information without interdependence on time and space. Nonetheless, based on the results of the study and the growing health issue causes by fake news related to COVID-19 on social media, we concluded that there is a need for social media users to double check the credibility of the content before posting the contents on social media. Individuals can check the source of information before reading or sharing the news content. Therefore, we recommended that social media users verify the authors, conduct an in-depth analysis of a news story by checking the times, review documentation to validate sufficient facts and statistics, identify fake photos, investigate other websites, and consult with practitioners while in doubt.

## Data Availability Statement

The raw data supporting the conclusions of this article will be made available by the authors, without undue reservation.

## Author Contributions

CZ: conceptualization and writing. TC: writing and methods support. AA: methods and analysis. All authors contributed to the article and approved the submitted version.

## Conflict of Interest

The authors declare that the research was conducted in the absence of any commercial or financial relationships that could be construed as a potential conflict of interest.

## Publisher’s Note

All claims expressed in this article are solely those of the authors and do not necessarily represent those of their affiliated organizations, or those of the publisher, the editors and the reviewers. Any product that may be evaluated in this article, or claim that may be made by its manufacturer, is not guaranteed or endorsed by the publisher.
